# A Review of Players’ Characterization and Game Performance on Male Rink-Hockey

**DOI:** 10.3390/ijerph17124259

**Published:** 2020-06-15

**Authors:** António Ferraz, João Valente-Dos-Santos, Hugo Sarmento, Pedro Duarte-Mendes, Bruno Travassos

**Affiliations:** 1 CIFD, Sports Research and Training Center, Jean Piaget University of Angola, Luanda 2177, Angola; antferraz@hotmail.com; 2 CIDESD, Research Center in Sports Sciences, Health Sciences and Human Development, Department of Sport Sciences, University of Beira Interior, 6201-001 Covilhã, Portugal; 3 Kinesiolab—Laboratory of Human Movement Analysis, Institute of Piaget, 2805-059 Almada, Portugal; 4 Faculty of Physical Education and Sport, Lusófona University, 1749-024 Lisbon, Portugal; j.valente-dos-santos@hotmail.com; 5 Research Unit for Sport and Physical Activity, Faculty of Sport Sciences and Physical Education, University of Coimbra, 3004-531 Coimbra, Portugal; hugo.sarmento@uc.pt; 6 Department of Sport and Well Being, Polytechnic Institute of Castelo Branco, 6000-266 Castelo Branco, Portugal; pedromendes@ipcb.pt; 7 Sport, Health and Exercise Research Unit—SHERU, Polytechnic Institute of Castelo Branco, 6000-266 Castelo Branco, Portugal

**Keywords:** sports physiology phenomena, anthropometry, body composition, performance analysis, injuries

## Abstract

The aim of this study was to review the evolutionary tendencies of research regarding to the study of male Rink-Hockey players´ and game performance. A systematic search was conducted in PubMed, Web of Knowledge and Scopus databases according to PRISMA method. The initial search identified 815 titles, resulting in 19 articles being included within the review. Original papers (English language) contained relevant data regarding rink hockey players’ performance or morphological/physiological demands, anthropometry/body composition characteristics were eligible. Studies were classified into categories: (1) Physiological Demands, (2) Anthropometry and Body Composition, (3) Game Characterization/Patterns, (4) Injuries. Results indicated that Rink hockey requires high intensity effort which demands both short and long duration efforts requirements from players. Body composition analysis shows to be an important monitoring tool which complements the understanding of the athlete’s cardiac adaptation. Game patterns shows a combination of specific game momentums with different outcomes according to the game zone. The intense short-term movements, collision and contact between players, in addition to the weight and speed of the hard ball and the stick, can considerably increase the risk of moderate and severe injuries. Lack of literature in Rink-Hockey is remarkable, and research is mainly focused on children and adolescents’ players. Furthermore, the existing research with adult elite athletes was assessed with a small sample size.

## 1. Introduction

Rink-hockey (also known as hardball or roller hockey) is an indoor intermittent team sport that requires the combination of short and long duration efforts and is considered a highly specialized and physiologically demanding sport [1]. Players move themselves on the field with specialized four wheeled quad skates, combining high-intensive effort while manipulating a wooden stick and a hard rubber ball [2]. The game is performed on a 20 m × 40 m rink with five players (one goalkeeper) and a wall around the rink perimeter [2]. Play time varies according to the athlete´s category—(1) 30 min, divided in two periods of 15 min (under-15s categories); (2) 40 min divided on two periods of 20 min each (senior male, senior female, under-23s male, under-19s male, under-18s female, and under-17s male); (3) international and national competitions can play a maximum of 50 min game time (two periods of 25 min each). In all the above-mentioned categories, there is a 10 min interval between the end of the first period and the start of the second period [3]. The game structure consists of intervals of different intensities such as short, middle, and long distances [4]. 

Regardless of rink-hockey being popular worldwide and played on five continents, there seems to be a lack of published scientific research [5], with only a small amount being published in English when compared with other team sports which are intensely studied and published such as football [6] or basketball [7]. 

Despite the lack of scientific literature, it is well reported that rink-hockey is a high intensity intermittent sport in which non-continuous actions of different speed levels are followed by incomplete recovery periods [8,9], requiring a well-developed metabolism for short and long duration efforts [8]. Also, intra- and inter-muscular and neuromuscular coordination are determinant for skating and for ball mastery and control using the stick [9]. In general, despite the specific requirements of the use of skating to move on the field, rink-hockey demands seem to be similar to other elite indoor team sports [10]. Accordingly, the improvement of specific conditioning traits of performance as strength, power, agility, speed, and maximal oxygen uptake is of particular importance for athletic performance [2]. 

The understanding of physical and physiological demands is essential for game success, however, it is also crucial to complement athletes’ fitness traits and game characterization. As in any other invasion team sport, game analysis, and more specifically, the analysis of technical and tactical behaviors of players and teams, it is fundamental to characterize the behavioral demands of the game [11]. To our best knowledge, with the exception of Sousa et al. [6] who analyzed the offensive play on the performance of rink-hockey goalkeepers, no other study has been developed with the purpose to characterize the technical and tactical game demands of rink-hockey. Further research is required in rink-hockey regarding performance analysis—specifically, the understanding of technical and tactical game demands [12].

There are constantly new approaches to improve team sports performance, such as tactical, technique, and physical conditioning [13]. Further research should be developed to improve the understanding of several indicators from training and game environment. Rink-Hockey must not be an exception. 

Summarizing, in the last 10 years, an increase in the number of studies has been observed in rink-hockey [14]. However, no research has been developed in order to clearly identify the players’ characterization and game performance on male rink-hockey. Therefore, the purpose of this article was to develop a systematic search and organize the literature of rink-hockey of the past two decades as an attempt to identify the evolutionary tendencies of the characterization of players and game performance on male rink-hockey. Particularly, it was our purpose to (a) identify the physiological and functional demands of rink-hockey; (b) identify and characterize the anthropometry, body composition, and conditional profile of rink-hockey players; (c) to identify the game patterns that characterize the game; and (d) to identify the prevalent injuries in this sport.

## 2. Methods

### 2.1. Search Strategy: Databases and Inclusion Criteria

A systematic search was conducted on the electronic databases PubMed, Web of Knowledge, and Scopus, according to the recommendations from the preferred reporting items for systematic and integrative reviews and meta-analysis statement (PRISMA) [15,16]. A searched by relevant publications between the 1 January 2000 and 31 January 2020 using the keywords “roller hockey” and “rink hockey” both associated with “elite”, “ethnicity”, “heart”, “ice hockey”, “internal and external load”, “physiology”, “sports sciences” and “youth” were performed. The publications that were retrieved had to follow the following specific criteria: the publications (1) contained relevant data regarding athletes´ morphological or physiological demands, or anthropometry or body composition characteristics; (2) were related to rink/roller hockey performers; and (3) were written in the English language. Exclusion criteria applied if they (1) contained other sports performers; (2) included females; (3) did not contain relevant data about athletes morphological and physiological demands, anthropometry, and body composition characteristics. 

To increase research accuracy, two reviewers (António Ferraz, João Valente-Dos-Santos) separately screened citations and abstracts to identify the articles which would potentially combine the inclusions criteria, having registered the characteristics of each study, including the name of the authors, sample, procedure and results or main outcomes. In case of disagreement regarding the eligibility of the article, a third reviewer (Hugo Sarmento) was included in order to reach a final decision. After all the articles were screened, the categories of the studies were organized into specific topics according to their main research topic.

### 2.2. Quality of Studies and Data Extraction

The quality of the studies was assessed as recommended in Faber et al. [17] using the criteria for critical review forms in Law et al. [18] (16 items) with the purpose of identifying the studies in which the low-quality score could interfere in the results. The possible criteria for each item were 1 (meets criteria), 0 (does not meet the criteria), or NA (not applicable). Articles were assessed with regards to their purpose (item 1), relevance of background literature (item 2), appropriateness of the study design (item 3), sample included (items 4 and 5), informed consent procedure (item 6), outcome measures (item 7), validity of measures (item 8), significance of results (item 9), details of intervention (item 10), analysis (item 11), clinical importance (item 12), description of drop-outs (item 13), conclusion (item 14), practical implications (item 15), and limitations (item 16). Based on the guidelines of Faber et al. [17], a final score was calculated allowing to classify the articles as: (1) low methodological quality (≤50%); (2) good methodological quality (50%–75%); and (3) excellent methodological quality (>75%). A data extraction sheet (from Cochrane Consumers and Communication Review Group’s data extraction template [19] was adapted to this review’s study inclusion requirements and then tested on 10 randomly selected studies (pilot test). One author extracted the data, and another verified it. Disagreements were resolved in discussions between these two authors (António Ferraz, João Valente-Dos-Santos). To organize the results, the studies were classified into categories established according to the major research topics.

## 3. Results

### 3.1. Search, Selection, and Inclusion of Publications

**Figure 1:**
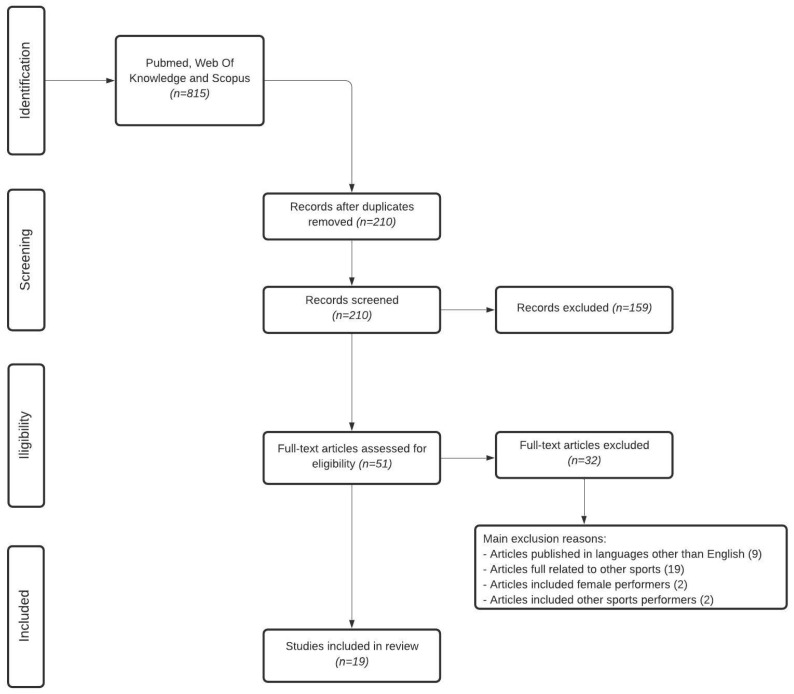
Flow chart of the article’s selection process.

The initial search identified 815 titles. These data were then exported to reference manager software Mendeley (Thompson Reuters, San Francisco, CA, USA). Duplicates (605) were eliminated automatically or manually. The remaining 210 were then screened for relevance based on their title and abstract information, with 159 being excluded. The remaining 51 were analyzed in more detail, and 32 papers were excluded according to the following criteria: (1) articles published in languages other than English (*n* = 9); (2) articles regarding other sports (*n* = 19); (3) articles including female athletes (*n* = 2); and (4) articles included others sports athletes with no relevant data (*n* = 2). Finally, 19 articles were included for the review. A flow chart of the study selection process is shown in Figure 1. 

The publication target years were limited to the past two decades, being from the year 2000 to January 2020. The remaining articles used for in depth reading revealed that there has been a surge of research available in rink-hockey. Included in this review 63.3% of the studies were published in the last five year (from 2015 to 2020), 31.5% from 2010 to 2014, and 5.2% before the year 2010. 

### 3.2. Quality of the Studies 

Regarding the quality of studies, as recommended in Faber et al. [17] and Sarmento et al. [6], the average score of the 19 selected quantitative studies was 85.9%. Three studies achieved 100%; 14 achieved an average rating of ≥75%; and only five achieved a score between 55% and 75%; none of the studies scored below 50%.

### 3.3. General Description of the Studies

**Figure 2:**
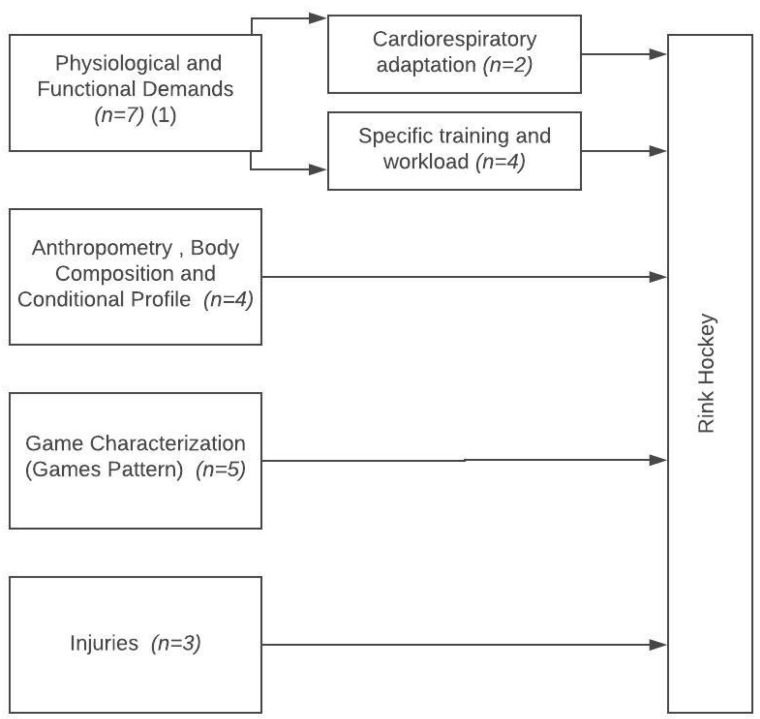
Flow chart of the main research topics in rink-hockey of the past two decades according to the inclusion criteria.

Studies were classified into the following categories established according to the main research topics that emerged from the content analysis: (1) physiological demands, (2) anthropometric, body composition and conditional profile, (3) game characterization and games patterns, and (4) injuries. See Figure 2. Studies were also identified by countries in which the studies were carried out—(1) Portugal (63.16%), (2) Spain (26.32%), (3) Germany (5.26%), and (4) Italy (5.26%).

The analyzed studies bring up information related to the characterization of specific competition level such as competitive demands of elite male rink-hockey and the influence of workload and well-being on athletes [1,20] or functional strength and shooting biomechanics analysis [8,20,21]. An insight of body composition, cardiac indicators, and peak oxygen uptake are described in adolescent players [22,23,24]. On the other hand, Coelho-E-Silva, et al. [22] present an approach model of sport selection under-17s in roller hockey combining anthropometry, functional and physiological information. Nutritional and body composition assessment in young Portuguese rink-hockey players was made by Silva and Silva [25]. Regarding game characteristics, observational instruments are explored to understand game patterns [14,26]. The high-intensity demands of the game combined with the mastery of skating and ball possession are described as potential injury risk [21,27,28].

### 3.4. Physiological and Functional Demands

Based on the analysis of the selected studies, the lack of research about this thematic in comparison with other team sports is obvious. However, Yagüe [1] in a study that involved six rink-hockey players, developed some field and laboratory tests. Also, it was possible to classify other studies in two sub-categories that allow for the characterization of physiological demands of rink-hockey—cardiorespiratory adaptations, specific training, and workload (see Table 1). 

### 3.5. Cardiorespiratory Adaptations 

The practice of sports offers clear opportunities to understanding cardiac remodeling [24]. Although there is limited information about cardiac indicators of adolescent hockey players, Castanheira et al. [24] compared 73 Portuguese players aged 16.4 ± 1.5 years old with other players with same age in other sports. Also, Valente-dos-Santos et al. [30] analyzed the importance of pulmonary function in rink-hockey players. Seventy-three Caucasian athletes were engaged on an incremental maximal test on a motorized treadmill for scaling peak oxygen uptake, while body size descriptors were also assessed [23] (see Table 1).

### 3.6. Specific Training and Workload 

Concerning the training environment, several factors can be considered especially relevant—therefore, the lack of conditioning coaches and the importance of multidisciplinary teams have been analyzed on both divisions in Spain (OK Liga and first division) [29]. On the other hand, rink-hockey researchers developed an investigation with 10 elite players which were tested on (1) time to exhaustion, maximum oxygen uptake, and running economy; (2) one repetition maximum bench press and half squat; (3) counter movement jump height; (4) 5 m, 10 m, and 20 m speed; and (5) 22 m agility. With the main goal of understanding the relationship between core strength and key variables of performance, athletes were also tested for (6) ventral, lateral-left, lateral-right, and dorsal core strength endurance using concentric-eccentric muscle tests [2]. Season training periodization is a complex process, mainly considering the implications of different training loads [20]. Therefore, variation of season workload and well-being among 10 professional rink-hockey players was assessed to understand the implications of load manipulation on regular (one match) and congested (two matches) weeks [20] (see Table 1).

### 3.7. Anthropometry, Body Composition, and Conditional Profile 

The studies that analyzed anthropometry and body compositions explored athlete´s profile to establish specific conditional profiles of rink-hockey players [22]. Also, the dietary intake, body composition and training data of child and adolescent rink-hockey players (38 children and 34 adolescents) has been assessed in order to compare body composition and nutrient deficiencies in Portuguese rink-hockey players [25]. Body descriptors such as body size, composition, and skeletal age seem to have a relationship between cardiac ventricular mass in under-17 players [30]. There is little information about elite adult athletes; however, when studying the relationship between power condition, agility, and speed performance, skinfolds and body composition information may be a determinant on the understanding of possible correlations [31] (see Table 2).

### 3.8. Games Characterization (Games Pattern)

Vaz et al. [32] analyzed the characterization of the biomechanics of the penalty stroke in rink-hockey with 15 rink-hockey players in order to measure the ball speed and the bend angle of the wooden stick at the moment of contact with the hard ball. More focused on game patterns of play, Sousa, Sarmento, Harper, and Valente-dos-Santos [5] and Sousa, Sarmento, Marques, Field, and Vaz, [14] developed studies focused on the analysis of the impact of goalkeeper behavior in opponent´s offensive play. Also, prominent tactical position [33] and determination of several combinations of possible behavior structure were analyzed in other studies with the goal to link behavior chain and the consideration of possible strategical interventions to improve players´ knowledge and resources [26] (see Table 3).

### 3.9. Injuries in Rink-Hockey 

A sample of 23 players (10 professional and 13 amateur) were assessed during two Spanish seasons (2014–2015 and 2015–2016) to analyze the injuries of rink-hockey players by considering the level of gravity vs. the level of competition [34], the results bring up an insight of 88 injuries and additional description according to the level of gravity vs. the level of competition [21]. In skating and during competition, sudden controlled turning and stopping can trigger the overuse of the adductor muscles and the whole surrounding region, causing groin pain [27]. The implications of groin pain were analyzed during Christiania stop (a sudden and intense change of direction and stopping) in professional rink-hockey players during a simulated test [27]. The influence of the knee joint position sense in rink-hockey players was studied, relevant information about the angular errors and benefits on proprioceptive accurate have been reported [28] (see Table 4).

## 4. Discussion 

### 4.1. Physiological and Functional Demands

The match demands of “multiple-sports” include periods of strenuous physical activity and recovery interspersed with brief periods of sprinting [35,36], but information about the impact of the external load is still sparse. Previous research has monitored the physiological indicators during rink-hockey competition, observing that the heart rate flows between 85% and 95% of the HR_max_ and blood lactate performance is between 4.0 mmol/l^−1^ and 4.6 mmol/l^−1^ [37]. In rink-hockey, there is not much research that includes the replication of match demands and the evaluation of player´s physiological profile. In other multiple-sprints sports, investigators have tried to replicate the demands by means of laboratory and field tests [38,39]. Our results showed that Yagüe et al. [8] tried to replicate the physiological demands designing a simulating skate test to describe the energy to extrapolate the results for the competition. Results obtained demonstrate that it is possible to achieve in laboratory physiological and metabolic demands. No significant differences were obtained in heart rate mean between stimulation tests and competition (189 ± 6.2 vs. 188 ± 5.6) [8]. In line with other studies, the authors revealed that the heart rate stays between 85% and 88% of HR_max_ during the whole game, remaining constant throughout the entire game [36,40]. Blood lactate during rink-hockey matches achieves values around 4.5–5.5 mmol/l^−1^ and 5.5–6.5 mmol/l^−1^ at the end of game, slightly lower than levels obtained during Yagüe et al. [8] in their simulated test (5.2 ± 01.0 mmol/l^−1^ in the middle and 7.2 ± 1.0 mmol/l^−1^ at the end). Maximal effort and maximum heart rate are frequently reached in matches, therefore Yagüe [8] mention that lactate levels during matches could be similar to those obtained in simulation tests. These reports are useful for monitoring and improving trainings by proposing the necessary changes. Nevertheless, new studies in rink-hockey matches are necessary, particularly those pertaining to blood lactate concentration immediately after high-intensity phases [8]. 

### 4.2. Cardiorespiratory Adaptations

It is recognized that sports training is related to the occurrence of concentric and eccentric cardiac hypertrophy [41]. Long-term intensive training promotes cardiac changes such as increased cavity diameter, wall thickness, and left ventricular mass (LVM) [38]. Although rink-hockey is not an endurance sport per se, it has been suggested, however that high values of cardiopulmonary function may be important to maintain a high level of activity during the entire game [4] and for efficient recovery from high-intensity short movements [39]. Regarding cardiac remodeling indicators in adolescent athletes, when compared with judo athletes, hockey players under-17 showed lager left auricle diameter (36.4 vs. 34.4 mm), interventricular end-diastolic septal thickness (8.1 mm vs. 7.5 mm) and left ventricular posterior wall thickness (7.7 mm vs. 7.1 mm) [24]. Regarding the scaling of peak oxygen uptake, male Rink-Hockey players aged 15.4 ± 0.6 years reached 3.89 L-min^−1^ [23]. Results suggest that variability of aerobic fitness in adolescent players may be influenced by tight volume (4.8 ± 1.0) and skeletal age (SA) (16.4 ± 0.6 years) [23]. There are few studies regarding maximal oxygen uptake in male elite players, but simulation tests and laboratory tests reports values between 50 and 60 mL/Kg/min [2,42]. Further research regarding cardiac morphophysiological traits and adaptations are required for a better understanding of the maximal oxygen uptake in elite rink-hockey players.

### 4.3. Specific Training and Workload 

Although few studies have addressed research focused on the characterization of morphological and physiological demands of roller hockey, it seems clear that in elite rink-hockey, as in other indoor team sports [10], enhancing specific variables of endurance like strength, power, speed, and agility seem to contribute to the increase in the players performance. Such results can be measured by time to exhaustion, maximum oxygen uptake, running economy during incremental testing [43], one repetition maximum in half squat and bench press exercises [44], vertical jump height in counter movement jump [45], and sprint times from speed and agility tests [46], which are important for athletic performance [2]. However, as in other sports, a relationship between core training and athletic performance is not consensual [2]. Nonetheless, similarly to results obtained in football [44], the level of total and ventral core strength-endurance was largely correlated with maximum oxygen uptake (r = 0.74 and r = 0.71, both *p* < 0.05) in elite male rink-hockey players, with a large correlation between the level of ventral core strength-endurance and the time to exhaustion (r = 0.66, *p* < 0.05) [2]. 

Monitoring athletes´ training loads is important to understand whether they adapt to the training program and to determine their feedback through fatigue [20]. The combination of different endurance determinants, such as internal load, fitness status and wellness, seem to have an important role in improving athletes´ global performance [47]. Coaches should periodize regular and congested weeks with different approaches to improve an athlete’s recovery [48]. Unfortunately, there is lack of literature about elite rink-hockey athletes. Regarding this fact, we believe that the development of more research involving athletes’ physiological traits and games load may be crucial to the update of new training environments. According to Cassassas et al. [29], coaches should take advantage of recent scientific knowledge concerning the implication of conditioning to improve training environment. 

In rink-hockey athletes, training sessions during normal weeks (one game) are associated with higher loads than sessions during congested weeks (two games) as indicated by differences in volume, rated of perceived exertions (RPE), and internal training load [20]. In terms of within-weeks variation, the day that is determined for more loads on normal weeks corresponds to the game day on congested weeks [20]. Therefore, it is suggested that there be an adjustment to the training load by decreasing volume, intensity, and consequently internal and external load of training tasks in congested weeks [20]. The characterization of external and internal load demands of elite rink-hockey players during competition and training is required to best characterize game demands of competition and consequent comparison with training sessions using indoor tracking system devices.

### 4.4. Anthropometry, Body Composition, and Conditional Profile

In the few last decades, several studies in different sports have focused their attention on the anthropometric characteristics of elite athletes [49,50] with the goal of identifying key determinants of performance that characterize specific sports and/or specific positional groups of players [9]. Therefore, the analysis of an athlete’s body composition is actually an important tool to characterize and improve sports performance [9]. Regarding to the anthropometry methodology used in the reviewed articles, in all studies body mass (BM) and stature was assessed. Additionally, in order to calculate the percentage of fat mass (FM) in two studies was used the sum of two skinfolds (triceps and subscapular) [25,30]. However, Silva and Silva [25] used the protocols of Frisancho [51], Valente-dos-Santos et al. [30], and the Slaugther et al. [52]. On the other hand, in the research of Coelho-e-Silva [22], the sum of four skinfolds (triceps, subscapular, suprailiac, and medial calf) was used following the standard procedures of Lohman et al. [53]. In all studies, a single observer completed all anthropometric procedures. Fat-free mass (FFM) was calculated as the difference between BM and body fat (BF) [12,25,30].

For example, the analysis of the energy intakes according to the demands of the competition on each sport, allows for an analysis of the necessary energy levels required by athlete´s for sport performance and especially for growth [2,29]. With this concern, Silva [25] characterized and compared the body composition and nutrient deficiencies between rink-hockey athletes and non-athletes. Results revealed that body mass index (BMI) was significantly lower in athletes than in non-athletes, mainly because of lower body mass. Similar results were observed in the analysis of 50 ice hockey players and controls [54], which corroborates that BMI is and indicator of body composition in athletes (athletes vs. non-athletes). Data revealed that mean intakes of carbohydrate and protein were adequate in both groups, but mean intakes of fat were above the recommended levels in athletes [25]. Significant differences were found for energy intake (EI) and exercise energy expenditure (EEE) between athletes and controls (*p* < 0.05), resulting in some cases of low energy availability in rink-hockey players. [25]. On the other hand, significant group differences (*p* < 0.05) were also observed for vitamin and mineral intakes in child and adolescent rink-hockey players due to higher mean intakes in control groups [25]. This fact may suggest that nutritional deficiencies in macronutrients and micronutrients may impair athletes´ growth and development promoting negative impact on their athletic performance. It has been reported that high body mass in athletes from contact sports may help in generating power and force, which in rink-hockey may be important for effective skating and physical contact, however, collision sports (where rink-hockey is also included) are likely to be favorable to athletes with greater levels of fat free mass rather than a greater total body mass [55]. The importance of body composition analysis extends to cardiac structure and performance [30]. Among adults, there has been a variety of methods to normalize left ventricular mass to body size, such as dividing left ventricular mass by body size variable namely stature [56]. However, body proportion change with growth and maturation, therefore, the relationship between cardiac size and stature may differ at different stages of development [57]. Well-trained adolescent rink-hockey players aged 15.4 ± 0.6 presented 55.6 ± 7.9 kg of fat free mass (FFM), and 11.8 ± 6.2 kg of fat mass (FM) [23]. Such results are in line with previous studies [58,59] and may be an important indicator of athletes’ body composition at this age. 

The indicators of FFM and FM, were simultaneous robust determinants of left ventricular mass (LVM) [58,59]. However, in the study by Valente-dos-Santos et al. [30], skeletal age was the variable that better relates to the left ventricular mass of roller hockey players. Upper body length measured by sitting stature was the most robust individual determinant of LVM. Sitting stature and fat mass was the most robust individual determinant of LVM normalized [30]. 

According to Coelho-E-Silva et al. [22], the comparison between under-17 male rink-hockey players selected for the Portuguese national team and athletes from regional level of the same age revealed advanced skeletal maturation and taller values with less subcutaneous adiposity of players selected for the Portuguese national team [22]. Data shows that international players are on average, 4.4 cm taller and 4.3 kg heavier than the local players; however, the body mass was not statistically significant [22]. In the end, the results revealed that differences in body size influence the performance by international players in hand-grip strength [22]. Authors concluded that young athletes who possess superior skills and functional abilities are nearest to becoming elite athletes [60]. Furthermore, when they present advanced biological maturity based on anthropometric determinants, they suggest an obvious motivation of sport performance [61].

Studies in adult male rink-hockey players are limited, and thus, those that exist consist of a small number of participants.

In rink-hockey, power, strength, speed, and agility are physical qualities of extreme relevance for the physical performance of the players, as in ice hockey [62]. Agility is described as a rapid movement of the whole body with the change of speed or direction in response to a stimulus [46]. A study conducted among 10 male rink-hockey players aged 14.20 ± 0.57 years with 58.62 ± 8.78 kg (body mass), 165.72 ± 8.45 cm (height), 21.26 ± 1.52 kg/m^2^) (BMI), and a sum six skinfolds of 51.80 ± 14.91 mm described that a significant inverse correlations exists between vertical jumps, and linear velocity in skating (countermovement jump vs. speed, r = –0.78) [31]. The same type of correlation exists between countermovement jump and agility teste (T-Shape tests) [31]. The results suggest that rink-hockey athletes who jump more, tend do have better agility traits [31]. Additionally, the vertical jump test looks to be a good predictor of linear speed and agility among talented young rink-hockey players [31]. Consequently, we believe that plyometric training may have an important role concerning the improvement of athletes’ speed and agility. 

### 4.5. Games Characterization (Games Pattern)

Rink-hockey is a particular invasion team sport [12] because it is possible to play behind the goals [33]. Cooperation between teammates is a dependent factor for success, which characterizes the internal logic and tactical behavior of players and teams [63]. It has been observed that the above-mentioned topics lack literature pertaining to match analysis [7]. As an exception, the analysis of the offensive process of an international U-20s team in five matches revealed 408 units of attacks (in circulation—indirect attack) followed by counterattack and quick transition. [64]. During the offensive process, major units of attack cover both lateral sides of the field and the central zone [64]. According to this match analysis, the central zone seems to be a determinant for the attacking movement, generating a greater number of shootings [64]. Per unit of attack a mean value of 2.73 passes were registered, showing how fast the offensive unit can be [12]. Regarding prominent tactical position, other research revealed that elite players actions are dependent of their positions [33]. Defenders and forwards received more balls from teammates and at the same time performed more passes [33] to other players´ positions. In opposition, wing players revealed the lower number of passes over competition [33]. It appears that players ‘positioning influences ball circulation across different game zones. The identification of patterns of play [26] supporting the following game actions—(1) shooting actions, (2) technical-tactical actions, (3) goalkeeper actions, and (4) other incidents—were assessed [18,20,31]. Results revealed that technical and tactical actions are involved in longer patterns of play in which behaviors do not have more than three links between the above-mentioned possible actions [26]. Regarding the offensive process, the shooting pattern is initiated with muscular power movement demanding a huge amount of muscle energy in which the athlete reaches a specific momentum while trying to stand completely balanced just on one foot [32]. As opposed to a shot (that can easily reach 100 km/h) [32], it is the goalkeeper who is usually the last line of the defense, wearing a protective gear in a crouched position [5]. The rink-hockey goalkeeper has an important role on his teams’ success. Concerning their influence on the fine line between winning and losing, Sousa et al. [5] developed an observational instrument tool that could help coaches to analyze the activity of rink-hockey goalkeepers. It is suggested that this type of tool may influence goalkeeper performance. 

As in futsal, rink-hockey is predominantly offensive and of finishing actions, players may reach 408–422 displacements during a game [35,65]. Nearly 50% of offensive actions lead to an attempt to score, however just 3% of attempts end in a goal [66]. It is known that opponents attacking, impact goalkeeper´s performance [14]. The variation of shooting zones influences the performance of the goalkeeper such as; (1) shots performed in the opposition defensive area, goalkeepers are 66% less likely to save the shot, in comparison with (2) shots performed in the intermediate area [14]. In addition, the variable “match status,” also seems to influence goalkeeper performance as they are more effective during the first half rather than the second, suggesting that this may be related with their lower physical conditioning [14]. The understanding of patterns may be crucial to develop new training strategies regarding the improvement of goalkeeper’s performance and field players. Future studies should characterize, by using specific technology, the internal and external load of different players position and different players behavior during an entire game.

### 4.6. Injuries in Rink-Hockey 

Rink-hockey is an intense collision and contact sport between players; in addition to the weight and speed of the hard ball and the stick, the risk of moderate and severe injuries can be considerable [34]. Few studies have been developed to show the incidence of rink-hockey injuries. Previous studies have been focused only in the number and severity of injuries [67,68]. According to some authors the type of injury which is more frequent seems to be head injuries [34,67]. In the Spanish league, 88 injuries were analyzed (professional vs. amateur), and the results reported a severity of mild 15.9%, moderate 50% and serious 30% [34], yet, the most frequent injuries were muscular by traumatic mechanism affecting the upper extremities as well as the head. [34]. The mechanism of injury coincides with the demands of a contact sport [67]. Also, it seems that the level of competition influences the level of injury. In professional competitions injuries, are mainly moderate (65% vs. 37.5%), while in amateur play, these are much more serious (45% vs. 20%) [34]. This context may be associated with the lack of specific training regarding injury prevention at amateur level [29]. On the other hand, the use of helmets for field players may significantly decrease head injuries and should be recommended as a preventative strategy.

One of the most common chronic injuries is promoted by the impact of the Christiania stop, which induces groin pain. [27]. Kinematics data refers that during the repetition of Christiania stop, athletes with no-groin pain show higher hip flexion, hip rotation, pelvis rotation, and knee flexion when compared with the groin pain group [27]. Players with previous groin pain tend to activate adductors the most, therefore the pain perception may lead to an adjust of task to avoid fear, leading to dysfunction of tasks [27]. On the other hand, results from the study of knee joint position of rink hockey players, suggest that athletes have higher proprioception indicators than non-athletes which contribute to injury prevention and enhanced performance [28]. This biomechanical information may lead to specific training strategies (from conditioning-strength coaches and physiotherapists) to reduce pain, injury prevention, and improved physical performance [29]. A more systematic study is required to link the type and occurrence of injuries with players’ characteristics, training, and competition load, type of competition, or even time between matches (congested vs. non-congested periods) 

## 5. Conclusions

In general, the results revealed that high values of adapted cardiorespiratory function of players may be important to keep elevated levels of activity during the entire game and for efficient recovery from high-intensity short movements. As expected, it seems that the development of performance, such as strength, power, speed, agility, flexibility, and maximum oxygen uptake, may increase athlete’s conditional performance profile. Characterization and comparison of body composition reveals to be an important monitoring tool of players’ development and energy intakes. Further research is required to understand the relationship between players’ characterization and performance over the rink-hockey game. The analysis of external and internal load of players as well as the analysis of technical and tactical actions should be promoted in order to identify reliable key performance indicators that help researchers and coaches to improve preparedness of players to the demands of the game.

The analysis of game patterns, shows a combination of specific game momentums with different outcomes according to the game zone. Their understanding may be crucial to develop new training strategies regarding the improvement of the goalkeeper and field players’ performance. In line with previous comments, further research is required to improve the understanding of game dynamics linking technical and tactical behaviors of players and teams. 

Regarding injuries, the intense short-term movements, collision, and contact between players, in addition to the weight and speed of the hard ball and the stick, can considerably increase the risk of moderate and severe injuries. Finally, there is a need for more research at elite levels regarding patterns of conditional profiles and physiological adaptation while, at the same time, understanding how demanding a top-level competition game can be. Therefore, we believe that it is important to understand what external load challenges the rink hockey athletes are subject to, aiming for the improvement of the training environment. 

Finally, only English articles searched in Scopus, Web of Knowledge, and PubMed were included in this study. Therefore, the lack of potentially relevant articles in other languages may be a limitation per se. Future steps should allow to focus the inclusion criteria in specific research topic. Additionally, we believe that the lack of international publications regarding the major topics reviewed in this paper may influence the actual reality of sport performance in rink-hockey. Therefore, there is a need to clarify the elite athletes’ traits and game demands in order to develop new evaluation and monitoring tools, which will improve rink-hockey training environments.

## Figures and Tables

**Table 1:** Articles predominantly related to physiological demands in rink-hockey.

Study and Country in Which Study Have Been Carried	Sample	Main Outcomes Measured	Results	Quality Score (%)
Valente-dos-Santos et al. (2013) [23]—Portugal	73 Portuguese, highly trained, male rink-hockey athletes, aged 16.4 ± 1.5	Anthropometry and peak oxygen fitness survey	The adolescents highly trained athletes chronological age (CA), 15.4 ± 0.6 years, skeletal age (SA), 16.4 ± 1.5 years; stature, 169.9 ± 6.9 cm; body mass, 63.7 ± 10.7 kg; thigh volume, 4.8 ± 1.0 L) performed and incremental maximal test on a motorized treadmill. Exponents for body size descriptors were 2.15 for stature (R^2^ = 0.30, *p* < 0.001) and 0.55 for thigh volume (R^2^ = 0.46, *p* < 0.001). The combination of stature or thigh volume and CA or SA, and CA^2^ or SA^2^, increased the explained variance in VO^2peak^ (R^2^ ranged from 0.30 to 0.55)	100
Yagüe et al. (2013) [8]—Spain	6 rink-hockey players aged 23.4 ± 3.1 years, 173.3 ± 4.7 cm and 72.3 ± 5.1 kg	Heart rate (HR), blood lactate, oxygen consumption, ventilation and respiratory exchanged ratio were recorded in a 20-metre multi-stage shuttle roller skate test, a tournament match, and a simulation test (ST)	Peak heart rate was 190.7 ± 7.2 heart.min^-1^. No differences in peak HR between the three tests. The mean of HR was similar between the ST and the match (86% and 87% of HR_max_ respectively). Peak and mean ventilation averaged 111.0 ± 8.8 L-min^−1^ and 70.3 ± 14.0 L-min^−1^ (60% of V_Emax_), respectively. V0_2max_ was 56.3 ± 8.4 mL-kg^−1^-min^−1^, and mean oxygen consumption was 40.9 ± 7.9 mL-kg^−1^-min^−1^ (70% of V0_2max_). Maximum blood lactate concentration was 7.2 ± 1.3 mmol/l^−1^. St yielded an energy expenditure of 899.1 ± 232.9 kj, and energy power was 59.9 ± 15.5 kg-min^−1^	81.3
Hoppe et al. (2015) [2]—Germany	10 elite rink-hockey players from the German National team (24.0 ± 1.5 years; 78.3 ± 8.2 kg; 178.6 ± 6.6 cm; 24.6 ± 1.7 IMC; 11.9 ± 2.9%^Body Fat^)	Athletes were tested in time to exhaustion, maximum oxygen uptake, and running economy; one repetition maximum bench press and half squat; counter movement jump height; 5 m, 10 m, and 20 m agility; ventral, lateral-left, lateral-right, and dorsal core strength-endurance using concentric-eccentric muscle tests	The level of core strength of total and ventral strength-endurance was largely correlated with maximum oxygen uptake (r = 0.74 and r = 0.71, both *p* < 0.05). There was a large correlation between the level of ventral core strength-endurance and the time to exhaustion (r = 0.66, *p* < 0.05)	93.8
Castanheira et al. (2017) [24]—Portugal	42 basketball players aged 15.32 ± 0.64 years, 73 rink-hockey players aged 15.29 ± 0.73 years, 28 judo athletes aged 15.23 ± 0.49 years, and 21 swimmers aged 15.35 ± 0.43 years	Anthropometry and echocardiographic exams were assessed by an experienced technician	Basketball and roller hockey players have larger left auricle diameters in comparison with judo athletes (F = 3.865; *p* = 0.011; ES-r = 0.316). Interventricular end-diastolic septal thickness (F = 7.287; *p* < 0.001; ES-r = 0.347) and left ventricular posterior wall thickness (F = 8.038; *p* < 0.001; ES-r = 0.362) of judokas are smaller compared to the mean value of other sports participants Relative left parietal ventricular wall thickness is lower among swimmers in comparison with judokas (F = 4.127; *p* = 0.008; ES-r = 0.268)	81.3
Reverter-Masia et al. (2017) [21]—Spain	Physical conditioning coaches from Ok Liga (*n* = 14), elite teams) and roller first division (*n* = 16)	A Survey was administered to each physical conditioning responsible by means of personal interview to determine the use of technology associated to the control of strength training	There were differences between categories in the utilization of the encoder (21.4 vs. 12.5%) and of the Optojump (35.7 vs. 25.0%). The countermovement jump (CMJ) (33.9%) and the squat jump (SJ) (20.5%) were the two vertical jump height tests further used	56.3
Cassassas et al. (2018) [29]—Spain	Physical conditioning coaches from Ok Liga (*n* = 14), elite teams) and roller first division (*n* = 16)	Survey administered to the people responsible for the physical conditioning to characterize medical staff and physical conditioning coaches	80% of the physical conditioning coaches of the OK Liga have degrees in Physical Activity and Sports Sciences against 40% of the first division teams (*p* < 0.001). Respectively, 45% and 20% had a Master and Doctoral degree in related fields of human performance. More than 80% of the OK Liga had physiotherapist, doctor and physical trainer significantly higher (*p* < 0.001) than first division teams (<45%). The percentage of the teams with kit men is also higher on OK Liga (>83.5% vs. 62.4%, *p* < 0.001)	62.5
Gonçalves et al. (2020) [20]—Portugal	10 elite rink-hockey players (29.3 ± 4.8 years; 178.3 ± 6.4 cm; 78.0 ± 3.9kg) from the Portuguese 1st league division	Perception of fatigue, stress, delayed onset muscle soreness, and quality of sleep were recorded; afterward, the Hopper index (1–7) was constructed with the sum of the four subjective ratings. Rating of perceived exertion (RPE) was collected approximately 30 min after each training session using Borg´s CR-10. Volume of training was also registered, and the session calculated	Players spend less time training on congested weeks (more than one game in the week) when compared with normal weeks. Similar results were identified in both congested and normal weeks concerning the training process of the days classified as MD-3 (three days before a match). There were significant differences between days classified as MD-3 and MD-2. Higher values of internal load, RPE, and volume, were observed respectively	93.8

**Table 2:** Articles mainly related to anthropometric, body composition, and conditional characterization.

Study and Country in Which Study Have Been Carried	Sample	Main Outcomes Measured	Results	Quality Score (%)
Valente-dos-Santos et al. (2013) [30] —Portugal	73 Portuguese male rink-hockey players aged 14.5 to 16.5 years	Anthropometry, skeletal age (SA) by Fels method, and allometric modelling of left ventricular mass (LVM) assessed in accordance with recommendations of the American Society of Echocardiography	Rink-hockey players (chronological age (CA): 15.4 ± 0.6 years; SA: 16.4 ± 1.5 years) showed an eccentric remodeling of left ventricle (LV) structure within the reference range (i.e., 0.24–0.42), a dilated LV chamber, but no LVM increase. Exponents for body size descriptors were 2.69 for stature (R^2^ = 27%; *p* < 0.001), 2.49 for sitting stature (R^2^ = 37%; *p* < 0.001), 0.76 for FFM (R^2^ = 31%; *p* < 0.001), and 0.22 for FM (R^2^ = 26%; *p* < 0.001). The combination of size descriptors with CA and SA increased the explained variance in LVM slightly (26%–45%)	100
Coelho-e-Silva et al. (2014) [22]—Portugal	32 Portuguese international and 41 local under-17 (U-17) (14.5–16.5 years) male rink-hockey players	Athletes were considered in the context of discrimination by level of competition using training history, anthropometry, skeletal maturity, and laboratory and field tests	International players had less hockey experience (years) but had more practice sessions and match time (minutes) during the season. Results revealed that international players are advanced in maturity status (42% vs. 22%). Local players are shorter and attained better performance in the 25 m dash, while international players performed better in sit-ups, ball throw and 20 m shuttle run. Fatigue index stemmed from Wingate anaerobic test was higher in local. There seems to have an interaction amongst strength, anaerobic fitness and training plus game time as a factor of discriminating international from local level players	100
Silva, Silva (2016) [25]—Portugal	72 male rink-hockey players (38 children and 36 adolescents) and 79 male controls (43 children and 36 adolescents). Athletes spent a mean of 8.5 ± 3.5 h of physical exercise per week (training and matches), while controls spent a mean of 2.0 ± 0.5 h of physical activity per week	Evaluation of training data, medical history, body composition, and typical dietary intake	Lower body fat (BF) and higher fat-free mass (FFM) were significantly observed in Rink-Hockey players. Intakes of carbohydrate and protein were adequate in athletes, on the other hand, mean intakes of fat were above the recommended levels. Differences were found in the energy intake (EI) and energy expenditure (EEE) between athletes and controls (*p* < 0.05), impacting in some cases of low energy availability in Rink-Hockey players. Intakes of vitamins and mineral intakes in child and adolescents were significantly higher (*p* < 0.05) in controlled groups. Low intakes of vitamins D, E, and K; calcium; iron; boron; and magnesium were reported in athletes, with the exception of thiamine *(p* = 0.449), riboflavin (*p* = 0.246), pantothenic acid (*p* = 0.065), magnesium (*p* = 0.061), and phosphorus (*p* = 0.051) in children and for niacin (*p* = 0.652), vitamin D (*p* = 0.406) and zinc (*p* = 0.783) in adolescents	81.3
Ferreira et al. (2019) [31]—Portugal	10 male roller hockey players with 14.20 ± 0.57 years old involved in the Portuguese national competition of under-15	Cross-sectional study. Strength was measured with squat jump (SJ) and countermovement jump (CMJ); sprinting time at 11 m, 22 m and 33 m was determined as well as in the agility t-test in roller skating	Significant inverse correlations were observed between vertical jumps and linear velocity in skating (−0.78). It was also verified a moderate inverse correlation between agility test with strength (−0.48). Lower limbs explosive and strength looks to be a strong predictor of skating linear speed and agility amongst young elite roller hockey players	87.5

**Table 3:** Articles predominantly related to the involvement of games characterization (games pattern).

Study and Country in Which Study Have Been Carried	Sample	Main Outcomes Measured	Results	Quality Score (%)
Mendo et al. (2002) [26]—Spain	Six observational sessions—six premier division matches involving a total of six different teams	Sessions were recorded for sequential game behavioral (technical-tactical actions, shooting actions, goalkeeper actions, other incidents) analysis	Sequential behaviors between technical-tactical actions, shooting actions were associated for definition of patterns of play. Information about game behavioral development that allow to link each behavior chain to strategies suited to improving players´ resources	68.8
Vaz et al. (2011) [32]—Portugal	15 athletes from different positions and levels (1st division—*n* = 7) (2nd division—*n* = 8) from the Portuguese league	Each athlete comprises three shot trials. Shots were recorded with a high-speed video camera (Photron Fastcam SA 2). A Stalker ATS radar (33.4 to 36 Ghz) was used to measure ball velocity	With no approach run, the ball speed may reach 90 ± 5.2 km/h (2nd league) and 102.0 km/h ± 4.6 (1st league). A shot with an approach run from a 1st league athlete may reach 115.4 ± 7.2 km/h. During contact with the ball, the angle of bending measured was 18.5 ± 3.2	62.5
Oliveira et al. (2015) [33]—Portugal	54 rink-hockey players from five different levels (U12 *n* = 10), (U14 *n* = 11), (U16 *n* = 10), (U18 *n* = 12) and Elite (*n* = 11)	Centrality metrics of the network of passes and analysis of the variance between competitive levels and tactical positions during three official matches.	No statistical differences in centrality levels of players between competitive levels. Tactical positions had a significant main effect on the central metrics. It was found that defender and forward position are the ones that receive the balls from the teammates more	75.0
Sousa et al. (2018) [5]—Portugal	64 coaches and 30 goalkeepers	Development and validation of and observational instrument using five methodological stages; (1) and (2) exploratory phase about rink-hockey; (3) and (4) development of a questionnaire and observational instrument; (5) test of the reliability of the instrument	The observational instrument was considered reliable to analyze the activity of rink-hockey goalkeepers. (Kappa of intra- and inter- observer were ≥ 0.80)	75.0
Sousa et al. (2020) [14]—Portugal	40 matches, including 1713 shots on goal from the Portuguese rink-hockey 1st division (2016–2017)	Data were analyzed by a specific notational analysis system developed and validated by Sousa et al. (2018) [5]. Additionally, a Chi-square test was performed to understand which variable was associated with the final action	Goalkeepers are more effective in the first half than the second half of matches. Goalkeepers performance is lower in the direct free-hits and penalties when compared with indirect free-hits. To save shots at the goal the most frequent technique used by goalkeepers is the “knee on the floor”. Results showed that when attacks commenced in the opposition´s defensive area, teams are 55% more likely to score and shots at the upper zones of the goal have higher probability of being successful	81.3

**Table 4:** Articles related to injuries in rink-hockey.

Study and Country in Which Study Have Been Carried	Sample	Main Outcomes Measured	Results	Quality Score (%)
Venâncio et al. (2016) [28]—Portugal	21 male roller hockey players (23.2 ± 4.2 years old; 81.8 ± 9.8 kg; 180.5 ± 4.1 cm) and 43 male voluntary participants (23.7 ± 3.9 years old; 85.0 ± 6.2 kg; 181.5 ± 5.0 cm)	Cross-sectional study which evaluated the knee joint position sense of the dominant limb, using a technique of open-kinetic chain and knee positioning	The results of this study show that the group of the roller hockey players presented significantly lower absolute (2.4 ± 1.2 vs. 6.5 ± 3.2, *p* ≤ 0.001) and relative (1.7 ± 2.1 vs. 5.8 ± 4.4, *p* ≤ 0.001) angular errors, in comparison with the non-athlete group	87.5
Reverter-Masia et al. (2018) [34]—Spain	10 professional roller hockey players (28.7 ± 6.78 years old; 74.9 ± 5.22 kg; 23.01 ± 1.57 IMC) and 13 amateur players (31.85 ± 8.81 years old; 82.69 ± 9.97 kg; 25.71 ± 2.58 IMC)	Descriptive analysis from injuries recorded during the 2014–2015 and 2015–2016 seasons	Of the 88 injuries registered, 15.4% were considered mild, 50% moderate, and 34.1% serious. The incidence of moderate injuries was higher in the professional in comparison to the amateur team players (65% vs. 37.5%), while serious injuries was superior in the amateur team (45% vs. 20%). The most frequent injury in both divisions was muscle-related by traumatic mechanism, mainly affecting the upper extremity	68.7
Vitale et al. (2019) [27]—Italy	8 male professional rink-hockey players—4 have had previous groin pain (27.75 ± 9.60 years old; 77.75 ± 5.50 kg; 179.75 ± 6.70 cm; 24.11 ± 2.04 IMC), and 4 with no-groin pain (23.25 ± 2.36 years old; 78.5 ± 5.07 kg; 177.5 ± 5.07 cm; 24.91 ± 2.01 IMC)	Prospective case series study was performed. Athletes were asked to perform the Chistiania stop, while muscle activity patterns and lower limbs kinematics were acquired with an optoelectronic system and infrared cameras allowing a computerized three-dimensional motion recording	Athletes from the groin pain experience, when performing Christiania stop, involuntary attempt to preserve the groin area. They showed lower peak values in kinematics parameters. The most frequent pattern of surface electromyography amplitude referred to adductor longus muscle, vastus medialis, tensor fascia latae and transversus abdominals. No-groin-pain group, the most frequent pattern of surface electromyography amplitude referred to transversus abdominis, adductor, vastus medialis and tensor fascia latae	87.5
